# West Nile Virus Infection in Pregnancy

**DOI:** 10.1155/2013/351872

**Published:** 2013-03-04

**Authors:** Robert D. Stewart, Stefanie N. Bryant, Jeanne S. Sheffield

**Affiliations:** Department of Obstetrics and Gynecology, The University of Texas Southwestern Medical Center, 5323 Harry Hines Bolevard, Dallas, TX 75390-9032, USA

## Abstract

A recent outbreak of West Nile virus has allowed for observations as to the clinical course of this emerging pathogen during pregnancy. We present three cases of West Nile virus infection during pregnancy. Case 1 presented at term with focal subjective weakness and fever. With supportive care, her symptoms were resolved within 7 days, and she subsequently delivered an unaffected term infant. Case 2 presented in the first trimester with fever and headache. Her symptoms were resolved in 8 days with supportive care. Case 3 was diagnosed during the first trimester during workup of nonspecific respiratory symptoms, with resolution of all symptoms in 24 days. Obstetricians need to be aware of the varied clinical presentation of West Nile virus during pregnancy.

## 1. Background

Since it was first detected in New York in 1999 [1], West Nile virus (WNV) has become an increasingly important pathogen in the United States. Most cases of WNV are asymptomatic; however, infection can result in febrile illness, encephalitis, meningitis, or poliomyelitis. The first case of intrauterine-acquired WNV was reported in 2002 [2], and in 2003 a case of maternal WNV encephalitis was reported [3]. In the summer of 2012, the United States experienced an epidemic of WNV, with 5,245 cases and 236 deaths reported to the Centers for Disease Control and Prevention. Texas has reported 1714 cases with 76 deaths thus far, with Dallas County being the most severely affected. According to the Texas Department of State Health Services, 400 cases of WNV illness and 18 WNV deaths have occurred in Dallas County to date. Here we report our experience with 3 cases of maternal WNV illness during pregnancy at Parkland Hospital.

## 2. Case 1

A 41-year-old G6P4A1 Hispanic female at 37-week gestation was presented to Labor and Delivery complaining of two days of bilateral lower extremity weakness with difficulty walking, fevers, and chills. She denied headaches, nuchal rigidity, nausea, vomiting, other weakness, or loss of sensation. Her prenatal care was complicated by diet controlled gestational diabetes. On arrival, she was febrile to 38.2°C with a pulse of 120. Her initial physical examination was otherwise benign with a normal neurological examination without any focal deficits. Strength was normal (5/5) in all extremities. Fetal heart tones were reassuring. Initial labs were negative for any obvious signs of infection and without leukocytosis. West Nile virus antibodies were drawn on admission. She became afebrile with acetaminophen and was admitted to the antepartum unit for further observation. On hospital day 1, she again complained of weakness and difficulty walking and was febrile to 39.0°C. Neurological examination remained unchanged, but, in light of continued neurological complaints with febrile episodes, the neurology service was consulted. Neurology confirmed a benign nonfocal neurological exam and agreed with infectious workup in progress. Later that night, she again was febrile to 38.3°C with a new complaint of several episodes of watery diarrhea. Stool WBC, O&P, culture and Clostridium difficile studies were sent. The next day she reported improvement in her subjective weakness; however, she was febrile to 38.6°C. Because of unclear etiology and episodic fevers, she was observed on Labor and Delivery with fetal monitoring for several hours that remained reassuring. She remained afebrile for the remainder of her hospital stay with continual improvement in symptoms. Her stool, urine and blood cultures were all negative. Her West Nile virus immunoglobulin (Ig)M resulted positive with negative IgG. She was discharged home on hospital day 5 after being afebrile with resolution of her lower extremity weakness. She did not keep her follow-up appointment in clinic.

At 38 weeks she presented to Labor and Delivery in active labor. She had an uncomplicated vaginal delivery of 2720 gm male infant, Apgar 8/9. On readmission her West Nile IgG was positive and IgM negative. Fetal cord blood was West Nile IgG positive and IgM negative. Infant serum on day of life 2 was WNV IgG positive and IgM negative. The infant's course was complicated by transient mild tachypnea that resolved on day of life 1 with no interventions. Maternal postpartum course was complicated by elevated blood pressures which required several doses of apresoline and initiation of amlodipine 10 mg daily on discharge. Since delivery, the infant has done well with no apparent postnatal effects. The patient was doing well at her postpartum visit with no apparent complications.

## 3. Case 2

A 29-year-old G4P3 Hispanic female at 10-week gestation was presented to the emergency department complaining of 4 days of fever and frontal-occipital headache with upper back myalgias. She had associated nausea but no vomiting. On presentation, she was febrile at 38.4°C and tachycardic at 110 bpm. On examination she had no neurological findings, her chest was clear, and her cardiac exam revealed tachycardia with normal rhythm. Her laboratory testing was normal. Respiratory DFA, influenza testing, and chest X-ray were normal. West Nile virus antibodies were obtained on admission. She was admitted for supportive care for a presumed upper respiratory infection. Her headache and fevers were treated with acetaminophen and she was given intravenous hydration. She was not initiated on antibiotics. On hospital day 1 her temperature rose to 39.1°C; however, her therapy was not changed and a repeat chest X-ray at this time showed no changes from admission. She remained intermittently febrile on hospital day 2, but had no new exam findings and reported subjective improvement of her symptoms, with resolution of her headache. By hospital day 4 she had remained afebrile for over 24 hours and had full improvement in her symptoms. She was discharged home with followup in our clinic. Nine days after discharge, her West Nile IgM and IgG both returned positive. She was followed up in our clinic sixteen days after discharge and reported full resolution of her symptoms. She is currently 14-week pregnant and without further complications.

## 4. Case 3

A 33-year-old G4P3 Hispanic female was presented to the emergency department at 12-week gestation, complaining of 2 days of difficulty breathing and a nonproductive cough, with associated subjective fever and chills, and one episode of emesis. She had no medical history and her prior pregnancies were uncomplicated. While in the emergency department, she was afebrile and mildly tachycardic at 100 bpm. Both cardiac and neurological examinations were normal; however, bilateral expiratory wheezing was present. Influenza testing, respiratory DFA, Tuberculosis immunoglobulin, throat culture, and chest X-ray were all negative for any abnormality. The remainder of her laboratory testing was normal. She was monitored in the emergency department where she received hydration and one albuterol nebulizer treatment with improvement in her symptoms. She remained afebrile with symptomatic improvement. A prescription for an albuterol inhaler was given, and she was discharged home with a suspected viral syndrome and associated reactive airway disease. At the time of discharge West Nile IgM and IgG were pending. Seven days after discharge her West Nile IgM and IgG both returned positive, and she was called to our clinic. At that time she complained of continued dyspnea, improved since her prior visit. She had no other symptoms and was afebrile with a normal exam, including no further wheezing. She was admitted for observation and symptomatic care with albuterol of which she received one treatment. On hospital day 1 her symptoms had resolved and she was discharged home. She was again seen in our clinic 7 days after discharge with a mild cough, for which she continued her albuterol inhaler as needed. She was afebrile with normal vital signs and an unremarkable exam. She returned seven days later, at which time she had full resolution of her symptoms. A sonogram was performed at 17-week gestation, showing a singleton fetus without any anomalies. She is currently at an estimated gestational age of 19 weeks and without further complications. 

## 5. Comment

WNV is a single-stranded mosquito-borne RNA flavivirus that is transmitted to humans through the bite of an infected mosquito. Approximately 80% of people with WNV remain asymptomatic. Of the 20% who develop symptoms, most develop West Nile fever, which consists of a febrile illness with an incubation period of 2–14 days. Clinical findings that accompany the fever are usually nonspecific and include malaise, anorexia, nausea, vomiting, myalgia, rash, and lymphadenopathy. Less than 1% of infected individuals develop WNV neuroinvasive disease, which can present as an aseptic meningitis or encephalitis. Clinical features of severe disease include fever, ataxia, optic neuritis, seizures, weakness, altered mental status, and myelitis. Risk factors for the development of severe disease include older age and immunosuppression. Diagnostic testing of WNV involves demonstrating West Nile specific IgM antibody in the serum of the infected individual. If West Nile IgM is present in the cerebrospinal fluid of an infected individual, it is diagnostic of WNV meningoencephalitis. There is no specific antiviral treatment of WNV, and care is generally supportive.

The obvious concern with WNV infection during pregnancy is the possibility of teratogenicity or adverse pregnancy outcomes. While intrauterine infection has been reported, with delivery of a fetus with bilateral chorioretinitis and cerebral abnormalities [2], a review of birth outcomes following maternal WNV infection in a national cohort did not demonstrate a significantly increased rate of adverse infant outcomes, including birth defects [4]. The rate of spontaneous abortion, preterm delivery, and low birth weight was no higher in the cohort of infected women compared to the general population. Of the 72 infants born of these women, only 3 possibly had WNV that could have been congenitally acquired although none had conclusive laboratory evidence [4]. Similarly, the study by Paisley et al. did not demonstrate any significant differences in infants of WNV seronegative versus seropositive women. They also found that the seroprevalence of WNV antibodies following an outbreak of WNV was 4%, with no infants having detectable WNV specific IgM antibodies [5]. However, given the limited reports of WNV infection complicating pregnancy, it is uncertain if WNV may have adverse effects on pregnancy outcomes. Therefore, more study is needed to determine the clinical effects of WNV on pregnancy outcomes.

There is also limited evidence as to the effect that pregnancy has on the clinical course of WNV infection. While mouse models have demonstrated that pregnancy increases the risk of severe WNV infection [6], limited evidence in human cases is available. The majority of currently available evidence is limited to case reports [2, 3], which are likely hampered by publication bias. A previous report of WNV encephalitis during pregnancy has been published, with a patient who became progressively obtunded and unresponsive after presenting febrile with meningeal signs. With supportive care she became responsive, but demonstrated residual lower extremity weakness [3]. A study of serologic evidence of WNV disease found that 32% of the seropositive women had a fever during their pregnancy, and 27% of seropositive women reported being told by a physician that they had WNV fever during their pregnancy. They did not report any WNV neuroinvasive disease in the report [5].

As there is little evidence describing the natural history of WNV in pregnancy, we offer our experience to help further describe the presentation of WNV during pregnancy. During the WNV season, we had a policy of aggressive screening for any pregnant woman who presented with neurologic symptoms or febrile illness. Table 1 shows selected characteristics of our cases of WNV infection during pregnancy. Only one of our patients presented with neurologic symptoms. The other two presented with a nonspecific illness, of which one was febrile. We recognize the possibility that case 3 may be an asymptomatic case of WNV that was incidentally found during the workup of a nonrelated illness. The duration of symptoms in our experience was 7–24 days. There was no residual neurologic sequelae in our series. The one infant born as of publication had no evidence of intrauterine infection. All three cases occurred remote from delivery, potentially decreasing the possibility of transmission to the fetus.

WNV is an epidemic infection in the United States, and while pregnancy does not appear to predispose to more serious infection, this issue is in no way resolved. Regardless of this, the potential consequences of WNV infection are significant and warrant aggressive screening of pregnant women who present with symptoms of infection, febrile illness, or neurological findings of unknown origin. The effect of pregnancy on WNV infection and the effect of maternal WNV infection on the fetus both require further investigation. As treatment is supportive, the best action is to prevent disease acquisition, by recommending pregnant women to wear protective clothing and use mosquito repellants.

## Figures and Tables

**Table 1:** Selected characteristics of West Nile virus infection during pregnancy at a single institution.

	Case 1	Case 2	Case 3
Maternal age (years)	41	29	33
Gestational age at presentation (weeks)	37	10	12
Fever	Yes	Yes	No
Headache	No	Yes	No
Nausea/vomiting	No	Yes	Yes
Neurologic symptoms	Yes	No	No
Duration of hospitalization (days)	5	4	1
Duration of symptoms (days)	7	8	24
